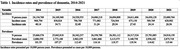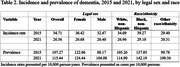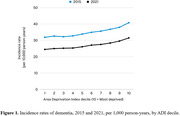# Trends in Incidence and Prevalence of Dementia Among United States Medicare Beneficiaries, 2014‐2021, by Race, Sex, and Neighborhood Socioeconomic Status

**DOI:** 10.1002/alz.093541

**Published:** 2025-01-09

**Authors:** Beau Blass, Cassie B Ford, Kim G Johnson, Amy G Clark, Samir Soneji, Richard J O'Brien, Bradley G Hammill, Emily C O'Brien, Jay B Lusk

**Affiliations:** ^1^ Duke University, Durham, NC USA

## Abstract

**Background:**

Estimating the incidence and prevalence of dementia is challenging. Cohort and brain banking studies can provide very precise estimates of incidence and prevalence in specific populations, but it is not clear how generalizable those estimates are to other populations. Furthermore, diagnoses of dementia made in clinical practice may differ from gold‐standard neuropathological diagnoses. Understanding trends in the incidence and prevalence of dementia as determined by routinely collected administrative claims data can provide critical context for clinicians and policymakers. Furthermore, understanding disparities in incidence and prevalence in the population at large can help to ameliorate biases from inequitable representation in survey and cohort designs.

**Method:**

We performed a retrospective study of 100% of Medicare claims from 2013‐2021 (2013 being reserved as a look‐back year). We defined incident dementia diagnoses in each year among patients without a known history of dementia based on validated international classification of diseases (ICD) algorithms. We defined prevalent dementia as patients with any prior history of dementia in each year. We stratified incidence and prevalence by race (classified as Black, non‐Hispanic, Other race/ethnicity, and White, non‐Hispanic), sex, and neighborhood socioeconomic status, measured by the well‐established Area Deprivation Index, which summarizes socioeconomic conditions at the census block group level (roughly 600‐1000 people).

**Result:**

From 2014‐2021, there were 5,721,711 incident cases of dementia in the United States. The average age at diagnosis was 80.5 years. Patients were 57.7% female, 86.6% White and 7.8% Black, 15.8% dually eligible for Medicare and Medicaid, and 41.5% from the South region. Table 1 shows incidence rates and prevalence of dementia from 2014‐2021. Table 2 shows incidence rates and prevalence stratified by legal sex and race/ethnicity. Figure 1 shows incidence rates of dementia stratified by neighborhood socioeconomic status.

**Conclusion:**

We found declining dementia incidence but increasing dementia prevalence in a sample that includes the vast majority of older adults in the United States. Differences in dementia incidence by sex, race/ethnicity, and neighborhood socioeconomic status remained notable in 2021. Our results have implications for health system performance in providing high‐quality and equitable clinical care for a growing population of patients with dementia.